# Automatic Prediction of Meningioma Grade Image Based on Data Amplification and Improved Convolutional Neural Network

**DOI:** 10.1155/2019/7289273

**Published:** 2019-10-01

**Authors:** Hong Zhu, Qianhao Fang, Hanzhi He, Junfeng Hu, Daihong Jiang, Kai Xu

**Affiliations:** ^1^School of Medical Information, Xuzhou Medical University, Xuzhou, China; ^2^Key Laboratory of Intelligent Industrial Control Technology of Jiangsu Province, College of Information and Electrical Engineering, Xuzhou University of Technology, Xuzhou, China; ^3^Affiliated Hospital of Xuzhou Medical University, Xuzhou, China

## Abstract

Meningioma is the second most commonly encountered tumor type in the brain. There are three grades of meningioma by the standards of the World Health Organization. Preoperative grade prediction of meningioma is extraordinarily important for clinical treatment planning and prognosis evaluation. In this paper, we present a new deep learning model for assisting automatic prediction of meningioma grades to reduce the recurrence of meningioma. Our model is based on an improved LeNet-5 model of convolutional neural network (CNN) and does not require the extraction of the diseased tissue, which can greatly enhance the efficiency. To address the issue of insufficient and unbalanced clinical data of meningioma images, we use an oversampling technique which allows us to considerably improve the accuracy of classification. Experiments on large clinical datasets show that our model can achieve quite high accuracy (i.e., as high as 83.33%) for the classification of meningioma images.

## 1. Introduction

Meningioma is a derivative of the meninges and spaces between the meninges, which is the second most common intracranial tumor, accounting for 13%–26% of intracranial tumors [1]. Most meningiomas are benign, slow growing, and surgically resectable. However, a small part is a malignant tumor, which has strong invasiveness and is easy to relapse after surgery [2]. It may lead to some symptoms including fading eyesight, vision loss, hemiplegia, epilepsy, etc., and severe cases may have the risk of sudden death. Meningioma poses a serious threat to people's health. There are three grades of meningioma according to the World Health Organization standards [3]. The preoperative grading of meningioma is extraordinarily helpful for clinical treatment planning and prognosis evaluation, which can reduce the recurrence rate of meningioma. At present, the method of grading meningioma is still pathology and imaging diagnosis combined with clinical experience, and pathology is the gold standard for meningioma grade classification. However, the way that pathology classifies meningioma grades takes a long time and is not efficient. Meningioma recurrence is important and possibly can be predicted with some certainty with preoperative MR image analysis [4]. In order to assist clinical practice and predict the recurrence of meningioma effectively, we use deep learning to predict the MR image of meningioma grades.

As a multilayer neural network learning algorithm, deep learning [5] learns not only nonlinear mapping between input and output but also the hidden structure of the input data vector [6]. It combines low-level features to form more abstractive high-level features to discover distributed representations of data [7]. Deep learning makes a significant breakthrough in face recognition, speech recognition, image recognition, and other fields. Tang et al. [8] proposed an end-to-end slice recognition method based on deep learning and prior knowledge to identify echocardiographic slices. Ying et al. [9] introduced a automatic classification algorithm based on deep learning for identifying criticality of chronic obstructive pulmonary disease from a large number of clinical samples. Yu et al. [10] used the deep learning technique to develop a nontraditional automatic algorithm for fetal facial recognition from ultrasonic standard section and obtained good result.

Convolutional neural network (CNN) [11, 12] is a popular deep learning model. It has the characteristics including local connections, weight sharing, and pooling operations, which can jointly lower the complexity of network and reduce the number of training parameters. CNN provides the model a certain degree of invariance for translation, distortion, and scaling and has strong robustness and fault tolerance, which makes it easy to train and optimize [13]. Al-Kadi [14] proposed a clinical decision support system that exploits the subbands' textural fractal characteristics for best bases selection of meningioma brain histopathological image classification to classify the four subtypes of grade I meningioma. The correct rate reached 94.12%. However, this system only classified the four subtypes of pathological sections in grade I meningioma and did not analyze MRI of meningioma for grades I, II, and III. Deepak and Ameer [15] used the concept of deep transfer learning to study the classification of glioma, meningioma, and pituitary tumors and used pretrained GoogLeNet to extract features from brain MRI images. Proven classifier models are integrated to classify the extracted features, and the average classification accuracy is up to 98%. Swati et al. [16] used pretrained deep CNN model and proposed a blockwise fine-tuning strategy based on transfer learning. The result can achieve average accuracy of 94.82% under five-fold cross validation. Although the above two models have high classification accuracy for brain tumors, they do not classify brain tumor grades. Yang et al. [17] used transfer learning to classify glioma MR images and achieved great results. However, this method requires the use of a rectangular region of interest segmentation for the tumor image in advance, which is inefficient.

Despite the tremendous success in the classification and diagnosis of brain tumors, there is still not much progress for automatic prediction of MRI meningioma grades. This is mainly due to the fact that the difference between meningioma grades is not very significant and needs to be evaluated by pathology, which is inefficient. Thus, we use convolutional neural network to assist in predicting meningioma grades to improve diagnostic efficiency. Due to the difficulty of narrowing down the critical features from as many as 1000 types of image features, such approaches cannot yield satisfactory results. To develop better techniques, we propose in this paper a new approach which combines an improved convolutional neural network with meningioma images to extract deep features for automatic classification. To resolve the issues of insufficient and unbalanced clinical image data, we also adopt an oversampling technique. It should be noted that grading of meningioma is only possible by means of neuropathological assessment (i.e., histology), and any analysis with any method (including deep learning) can only predict to some extent the grade and final tissue analysis-based diagnosis. The gold standard of meningioma diagnosis is still histopathological assessment, and the method we used in this paper is only used to assist the prediction of meningioma grade.

## 2. Meningioma and Classification Methods

The WHO distinguishes three histological grades (I, II, and III) and 15 subtypes of meningioma [18]. The vast majority of meningioma is grade I, benign tumor. The growth of such tumors is slow, recurrence after complete resection is rare, and prognosis is good. 20% to 25% of meningioma is grade II, atypical tumors, and 1% to 6% is grade III, malignancies [19]. These types of meningioma have strong invasive ability and characteristic of rapid growth and are easy to relapse after surgery [20]. Among the 15 subtypes, there are 9 WHO I subtypes (with lower risk of recurrence and invasiveness), 3 WHO II subtypes, and 3 WHO III subtypes (with higher risk of recurrence and invasiveness) [20] (see Tables 1 and 2 for details). To reduce the risk of meningioma recurrence, we use the improved LeNet-5 network to predict three grades of meningioma.

## 3. The Principle of Convolutional Neural Network Model

The LeNet-5 model we use is from Deep Learning Toolbox [21]. The underlying convolutional neural network (CNN) contains a feature extractor which consists of a convolutional layer and a pooling layer. At the input layer, a size-fixed image dataset is taken as input. In the convolutional layer, the input image is convolved by the convolution kernel to extract local features and the connections between the layers of network are reduced, which could potentially lower the risk of overfitting. In the pooling layer, the downsampling method is used to reduce the dimension and select the feature image. The convolution and pooling operations can be repeatedly applied according to the actual problem. Finally, the obtained result is fed into a fully connected layer of the CNN to yield a column vector, which contains the probabilities of various features, and represents the probability of each type. The one with the highest probability is taken as the final predicted type.

In the following sections, we discuss in detail each of the three layers of convolutional neural network (CNN): convolution layer, pooling layer, and fully connected layer.

### 3.1. Convolution Layer

Each neuron in the convolution layer is locally connected to the upper layer. The eigenvalue of the convolution layer is the result of dot-multiplication and addition of each pixel point and convolution kernel weight. Convolution kernel moves in a fixed step size to perform local feature extraction on all pixels of the upper layer image. Local connection reduces the number of network parameters and computational time complexity, which is conducive to network training of images. The mathematical expression of the convolution layer is

(1) $$\begin{array}{c} x_{j}^{n}=f\left(\underset{i\in Fj}{\sum }x_{j}^{n-1}\times k_{ij}^{n}+b_{j}^{n}\right), \end{array}$$

where *n*  is the number of layers in the convolution layer, *f*() is an activation function, *Fj* indicates the upper level feature map,  *k* is the convolution kernel,  *b* is a bias, *i* is the index of an input neuron node, and  *j* is the index of an output neuron node.

### 3.2. Pooling Layer

Pooling layer pools the feature map of convolutional output to reduce the dimensionality. It is mainly used for feature extraction from the convolutional features obtained in the previous layer. The mathematical expression of the pooling layer is

(2) $$\begin{array}{c} x_{j}^{n}=f\left(\beta_{j}^{n}\text{down}\left(x_{j}^{l-1}\right)+b_{j}^{n}\right), \end{array}$$

where *f*() is an activation function, down() is a subsampling function, *β* is the coefficient(s) of the subsampling layer, *b*  is a bias, and  *j* is the index of an output neuron node.

There are two types of pooling operations: max pooling and average pooling. The model uses the average pooling, whose basic steps are similar to those in the convolution layer. The pooling layer has two key parameters, filter size and fixed step size in its computation. It uses the maximum value pooling or average pooling to reduce the dimension of the convolution feature image and finally selects each pixel according to the size of filter and the size of step.

### 3.3. Fully Connected Layer

The fully connected layer in CNN is a common layer connecting the convolution layer and another common layer. It flattens the feature data from its parent (convolution and pooling) layer and uses an activation function to perform some nonlinear transformations. The obtained result is then used to classify the images.

Each neuron of the fully connected layer has the following output:

(3) $$\begin{array}{c} h_{w.b}\left(x\right)=f\left(w^{T}x+b\right), \end{array}$$

where *h*_*w*.*b*_(*x*) is the output value, *f*() is the activation function,  *w*  is the weight vector, *x* is the feature vector, and *b* is a bias.

## 4. Improvements of Convolutional Neural Network Model

### 4.1. Adding Softmax Layer

The convolutional neural network model used in the experiment is a LeNet-5 model in deep learning. After the full connection, the model is directly normalized using the sigmoid activation function, and the maximum value of the normalized probability value is taken by the max method as the final output result. All the grades of meningioma are classified in the original model, and the grades II and III could not be separated. Therefore, after the fully connected layer, the experiment is added with a softmax layer to improve the results.

The softmax function has the following form:

(4) $$\begin{array}{c} f\left(z\right)=\frac{e^{Zc}}{\sum_{d=1}^{C}e^{Zd}}\;, \end{array}$$

where vector *z* is the input data of dimension *c* and *f*(*z*) is the output vector of dimension *c*.

### 4.2. Data Amplification

**Figure 1:**
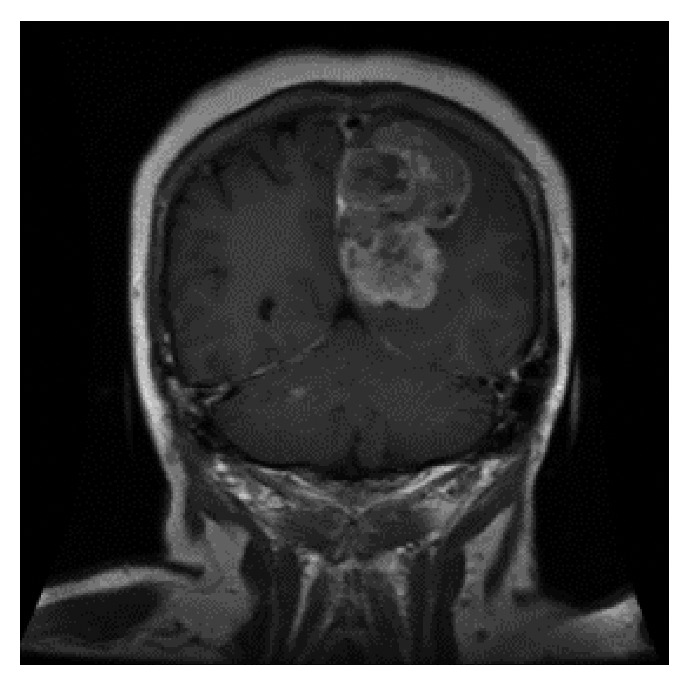
WHO III original image before oversampling.

Since some grades of meningioma are relatively rare, our study suffers from the problem of lacking sufficient original training sets, which could cause the network model to learn inadequately the necessary features of the images. To resolve this issue, we will use mirroring and rotation oversampling techniques to augment images of scarce grades to evenly distribute image data, while expanding the training dataset (Figure 1).

**Figure 2:**
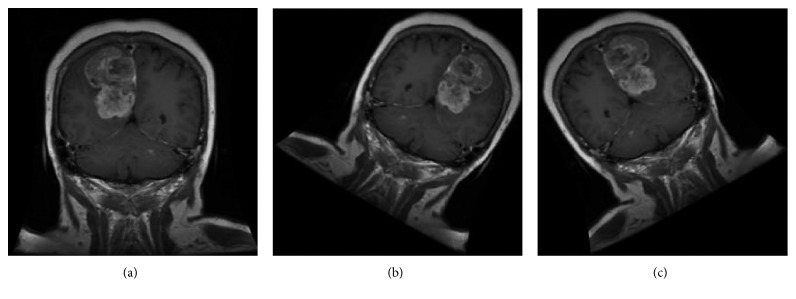
WHO III images after oversampling. (a) Mirror image. (b) Rotation image. (c) Rotation image.

We take the WHO III meningioma as an example to show the images before and after oversampling. For ease of presentation, we only select part of the images after oversampling. The specific images are shown in Figure 2.

**Figure 3:**
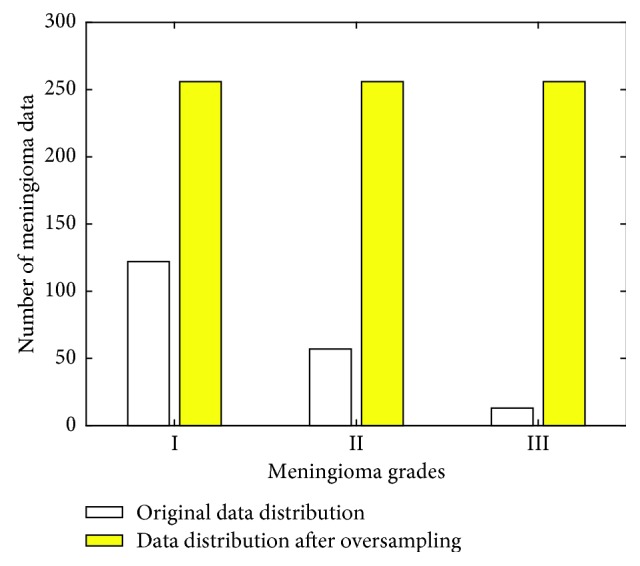
Distribution of meningioma data before and after oversampling.

The distribution of image data of meningioma is shown in Figure 3:

As can be seen from Figure 3, the original meningioma image data are less and the distribution is not balanced. After using oversampling technique, the amount of image data of the meningioma is increased and evenly distributed.

### 4.3. Improvement of CNN Architecture

**Figure 4:**
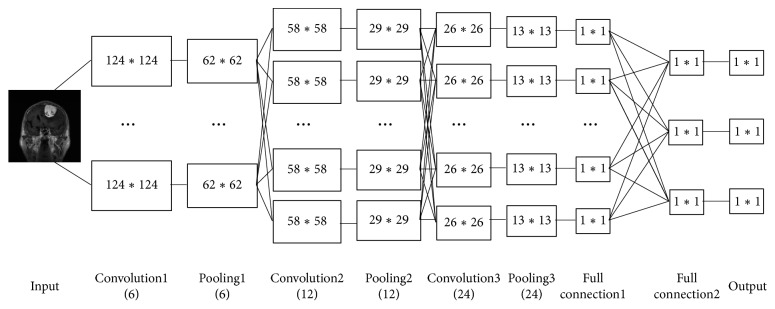
Improved network structure.

As a classic model, convolutional neural network is capable of achieving over 99% accuracy for MNIST handwriting recognition with a five-layer architecture. For the problem of meningioma classification, however, the original five-layer CNN architecture is no longer adequate for us to extract the necessary features. This is because the meningioma image is more complicated with much higher dimensions than the MNIST data. To further improve this model, we add additionally a convolution layer and a pooling layer to the basis of the original five-layer network. Accordingly, the network parameters are also adjusted. Particularly, we use 6 feature figures in the first convolution layer. The number of feature figures in the second convolution layer is increased to 12, and the convolution kernel size is changed to 4 *∗* 4 from 5 *∗* 5. For the newly added third convolution layer, we use 24 feature figures and 4 *∗* 4 convolution kernel size. Also, all filters are 2 *∗* 2 in size. The specific network structure is shown in Figure 4:

### 4.4. Iterative Descent Rate

Iterative descent rate is responsible for updating the weights and offsets during the training process. A change to this rate could affect the offset of the neural network after each round of training. Since the rate for classification, using the original iterative rate of decline, is very low, which indicates that the original offset cannot effectively update the network, we repeatedly adjust the iterative descent rate in our experiments and finally select an appropriate value for our problem.

### 4.5. Replacing the Original Activation Function

The original LeNet-5 network used the sigmoid activation function. The sigmoid activation function has soft saturation. It is easy to produce gradient disappearance during training and learning and is impossible to complete deep network training. The sigmoid activation function is not ideal for grading meningioma. Therefore, the experiment will replace the sigmoid activation function and apply the tanh activation function, ReLU activation function, and ELU activation function to the network, respectively. Finally, we select the appropriate activation function according to the final test results.

## 5. Experiment Analysis

### 5.1. Experiment Platform

The hardware and software environment involved in this experiment is as follows:

  Software environment: Windows 7 (64-bit) operating system, Matlab R2016b
  Hardware environment: Intel Core i5 6500-3.2 GHz, 4.0 GB RAM

### 5.2. Production of Medical Image Dataset

The meningioma dataset used in this experiment was obtained from the Affiliated Hospital of Xuzhou Medical University. What we need to declare is that meningioma dataset used in our study has followed all the procedures required by the Chinese government's law (similar to IRB). The data have been strictly reviewed by those in charge of such issues, and all sensitive information has been removed. This study is purely for research purpose and does not have any dispute of interest. A total of 222 MR meningioma images in Ocor (coronal) orientation were collected during the experiment. Among them, 192 images are used as the training set and 30 images as the test set. The gold standard of meningioma diagnosis is still histopathological assessment. The criteria for the labeling of meningioma dataset used in our experiments were all classified according to histopathology. The original MR images have size 512 × 512. To improve the efficiency, we set the image size in our experiment to 128 × 128.

In order to solve the problem of scarce data in meningioma training set, the experiment used image mirroring and rotation method in the preimage processing and finally expanded the training set to 768 and guaranteed the number of grades I, II, and III of meningioma.

### 5.3. Effect of Softmax Layer on Classification Results

**Figure 5:**
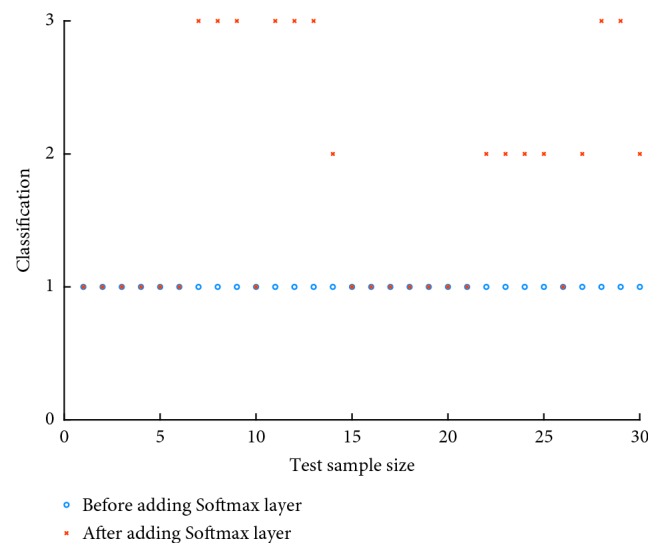
Original network classification. Classification of network after adding softmax layer.

Figure 5 shows the comparison before and after adding the softmax layer. The abscissa indicates the number of test sample size, and the ordinate indicates grades I, II, and III of meningioma. When the softmax layer was not added, the output results were of grade I; after adding the softmax layer, meningioma grades could be distinguished, which showed that the softmax layer is important for image classification of meningioma.

### 5.4. Activation Function

The sigmoid activation function is prone to gradient disappearance, which leads to the network not being able to update effectively. Therefore, this experiment compares the commonly used activation functions of tanh, ReLU, and ELU to select the activation function that is most suitable for the network.  Table 3 shows the impact of different activation functions on test results.

As can be seen from Table 3, when the network uses the ELU activation function, the test accuracy rate is the highest. The mathematical expression of ELU is

(5) $$\begin{array}{c} f\left(x\right)=\left\{\begin{array}{cc} \alpha \left(e^{x}-1\right), & x<0,\\ x, & x\geq 0, \end{array}\right. \end{array}$$

where *α* is a parameter, and in this experiment, we set *α* to 0.1. ELU combines sigmoid and ReLU, with soft saturation on the left side, which makes the ELU more robust to input changes or noise; the right side is nonsaturated, which makes it possible to alleviate the gradient disappearance and converge faster.

### 5.5. Comparison of the Original and Improved CNN Architecture

The original five-layer architecture has low accuracy in meningioma grade classification. To achieve better accuracy, we use the improved seven-layer CNN architecture in our experiment. Accordingly, we also use the improved size of convolution kernel, the number of feature maps, filter size, and iteration rate.  Table 4 lists the correct classification rate of meningioma in some five-layer networks.  Table 5 shows the effect of some changes on the error rate of meningioma grading. Figures 4 and 5 show the error distribution of the original architecture and the improved one.

**Figure 6:**
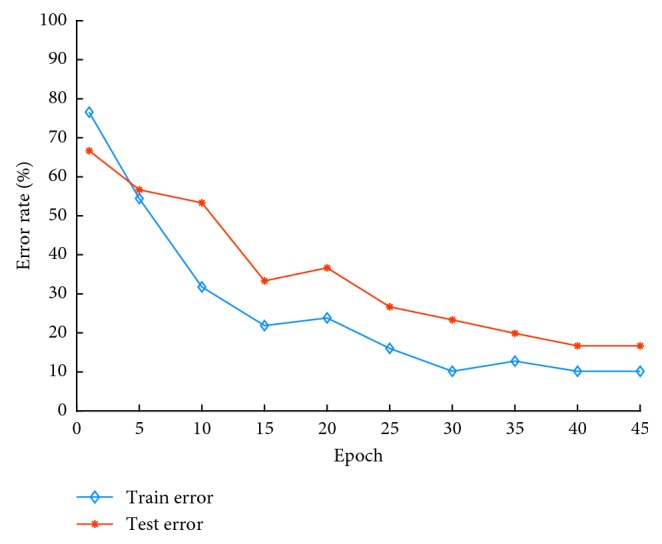
Error rate distribution.

As can be seen from Figure 6, when epoch is equal to 40, the network reaches convergence. The test set error rate is 16.67%, and the training set error rate is 10.16%.

**Figure 7:**
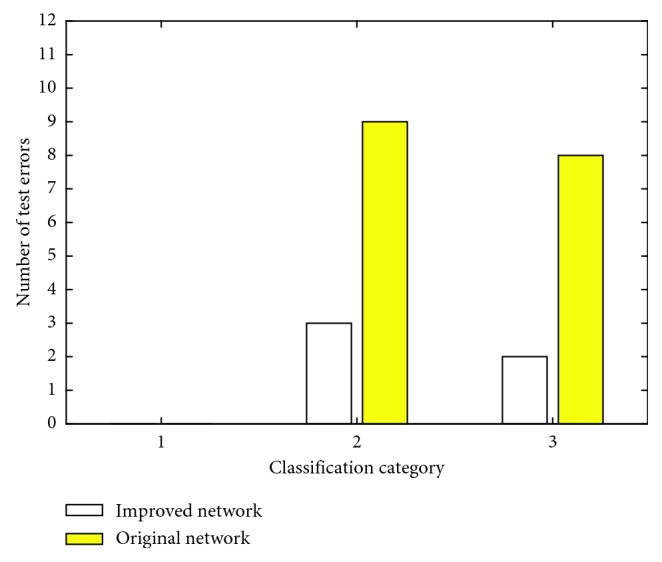
Comparison of error distribution before and after network improvement.

Figure 7 indicates a comparison of errors before and after network improvement. The abscissa indicates three grades of meningioma, and the ordinate represents the number of errors in each type. It can be seen from the figure that the original network has a poor classification of grades II and III of meningioma. After improvement, the error rate of grades II and III of meningioma can be reduced.  Table 6 shows the error location distribution table before and after the network improvement. In the 30 test samples, the meningioma grades are graded using the original network architecture, and finally 17 images are incorrectly graded. It is graded using an improved network architecture, with only 5 of the 30 test samples being incorrectly ranked. The experimental results show that the improved network structure classification accuracy rate is improved before the improvement.

### 5.6. Comparison of an Existing Model and the Model Proposed in This Paper

**Figure 8:**
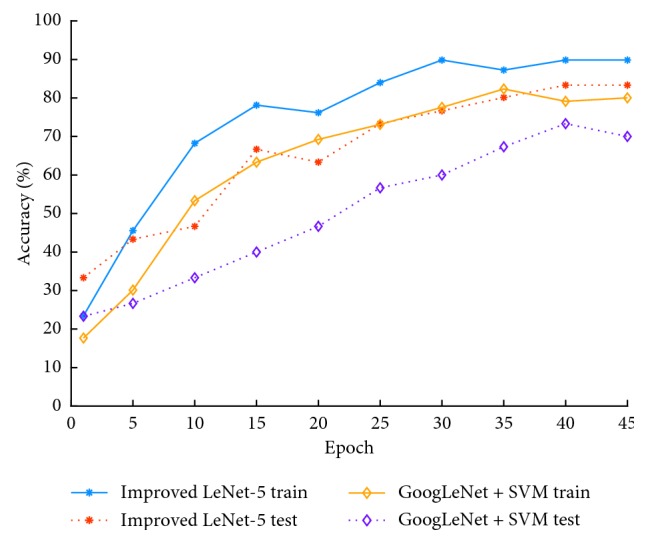
Comparison of an existing model and the model proposed in this paper.

We used the GoogLeNet model trained in ImageNet dataset of the literature [15] to grade the original meningioma dataset and compare it with the model of this paper. The results are shown in Figure 8.

Figure 8 shows the accuracy in the model of the literature [15] and the model of this paper on the grades of meningioma. It can be seen from the figure that the model in this paper (training accuracy rate 89.84%; test accuracy rate 83.33%) is better than the model in the literature [15] (training accuracy rate is 82.33%; test accuracy rate is 73.33%).

### 5.7. Wilcoxon Signed-Rank Test

In this experiment, we used the original network and the improved network to perform a 10-fold cross validation on the training set and recorded the accuracy of each verification. At the same time, we performed statistical tests using Wilcoxon signed-rank test for paired sample comparison. The specific table is shown in Table 7.

According to the Wilcoxon signed-rank test, the *P* value is 0.005 and less than 0.05, which is statistically significant. The results of the improved network have “clinical” significances.

## 6. Discussion

In this study, several experiments were designed to validate our method. Particularly, we first compared the effects of the softmax layer on the accuracy of meningioma grade classification and found that adding the softmax layer can achieve much better result. Subsequently, we expanded the training set and compared the four activation functions and found that the ELU function works best. Then, we compared the improved LeNet-5 architecture with the architecture in the literature [15] and found that the accuracy of our architecture is higher (83.33%), and we confirm that our architecture has an advantage in the prediction of meningioma grading. Finally, we used the Wilcoxon signed-rank test for paired sample comparison to perform ten statistics on the model and calculated that the *P* value was less than 0.05, which was statistically significant. Despite the achievements reported in this paper, several improvements remain possible: On the one hand, the data samples used in the experiment are still insufficient and it is easy to produce the phenomenon of overfitting. On the other hand, the performance of the improved model is still lacking and the accuracy of grading meningioma is still not high enough. Future research in the domain shall address these issues, possibly collecting new data and using a suitable generative adversarial network to augment data and further improving the model in this paper.

## 7. Conclusions

In this paper, we used the mirroring and rotation oversampling techniques to augment the meningioma dataset and improve LeNet-5 to assist in predicting the grades of meningioma. We increased the depth of the network and adjusted the network parameters to obtain deeper features of the image, while adding the softmax layer to distinguish the three grades of meningioma and changing the activation function to improve the accuracy of prediction. The results show that our proposed method can achieve rather high accuracy and has the potential to assist clinical diagnosis.

## Figures and Tables

**Table 1:** Histological subtypes and biological behavioral characteristics of meningioma with lower risk of recurrence and invasiveness.

Subtype	WHO classification	ICD-O code
Meningothelial meningioma	I	9531/0
Fibrous meningioma	I	9532/0
Transitional meningioma	I	9537/0
Psammomatous meningioma	I	9533/0
Angiomatous meningioma	I	9534/0
Microcystic meningioma	I	9530/0
Secretory meningioma	I	9530/0
Lymphoplasmacyte-rich meningioma	I	9530/0
Metaplastic meningioma	I	9530/0

**Table 2:** Histological subtypes and biological behavioral characteristics of meningioma with high risk of recurrence and invasiveness.

Subtype	WHO classification	ICD-O code
Chordoid meningioma	II	9538/1
Clear cell meningioma	II	9538/1
Atypical meningioma	II	9539/1
Papillary meningioma	III	9538/3
Rhabdoid meningioma	III	9538/3
Anaplastic (malignant) meningioma	III	9530/3

**Table 3:** Impact of different activation functions on network test results.

	Sigmoid	Tanh	ReLU	ELU
Characteristics	Gradient disappears	Convergence speed is faster than Sigmoid; gradient disappears	The input is positive, the gradient does not disappear; the input is negative, the gradient disappears.	It combines sigmoid and ReLU; and gradient disappears
Test accuracy rate	70.00%	56.67%	76.67%	83.33%

**Table 4:** Impact of original network structure on the error rate of meningioma classification.

Network layer	Feature map		Convolution kernel		Filter size		Iteration rate	Error rate
	Conv1	Conv2	Conv1	Conv2	Conv1	Conv2		
5	6	12	5 × 5	5 × 5	2 × 2	2 × 2	0.001	60.00%
5	6	12	5 × 5	5 × 5	2 × 2	2 × 2	0.0001	50.00%
5	6	12	5 × 5	5 × 5	2 × 2	2 × 2	0.0005	56.67%
5	6	12	9 × 9	5 × 5	2 × 2	2 × 2	0.0001	30.00%
5	8	16	9 × 9	5 × 5	2 × 2	2 × 2	0.0001	50.00%

**Table 5:** Impact of improved network structure on the error rate of meningioma classification.

Network layer	Feature map			Convolution kernel			Filter size			Iteration rate	Error rate
	Conv1	Conv2	Conv3	Conv1	Conv2	Conv3	Pool1	Pool2	Pool3		
7	4	8	16	5 × 5	5 × 5	4 × 4	2 × 2	2 × 2	2 × 2	0.003	83.33%
7	5	10	20	5 × 5	5 × 5	4 × 4	2 × 2	2 × 2	2 × 2	0.0001	53.33%
7	5	10	20	5 × 5	5 × 5	2 × 2	4 × 4	3 × 3	2 × 2	0.0005	60.00%
7	6	12	24	5 × 5	5 × 5	2 × 2	4 × 4	3 × 3	2 × 2	0.0001	23.33%
7	6	12	24	5 × 5	5 × 5	4 × 4	2 × 2	2 × 2	2 × 2	0.0001	16.67%
7	6	12	24	5 × 5	5 × 5	4 × 4	2 × 2	2 × 2	2 × 2	0.00005	16.67%
7	8	16	32	5 × 5	5 × 5	4 × 4	2 × 2	2 × 2	2 × 2	0.0001	26.67%
7	8	16	32	9 × 9	5 × 5	2 × 2	4 × 4	2 × 2	2 × 2	0.0001	56.67%

**Table 6:** Error location distribution before and after network improvement.

Network	Number of test sets	Number of test errors	Error distribution location
Original network	30	17	7, 8, 9, 10, 11, 12, 13, 14, 22, 23, 24, 25, 26, 27, 28, 29, 30, 10, 14, 26, 28, 29
Improved network	30	5	

**Table 7:** Statistics based on Wilcoxon signed-rank test for paired sample comparison.

Times	Original network accuracy (%)	Improved network accuracy (%)	*Z*	*P*
1	67.53	81.82	−2.804881	0.005
2	72.73	84.42		
3	68.83	87.01		
4	75.32	87.01		
5	70.13	85.71		
6	72.73	83.12		
7	68.83	81.82		
8	77.72	81.82		
9	72.37	84.21		
10	77.63	85.53		

## Data Availability

The data used to support the findings of this study are available from the corresponding author upon request.
